# Dermal Allograft Augmentation for Large and Massive Rotator Cuff Tears and Revisions: A Case Series

**DOI:** 10.7759/cureus.57090

**Published:** 2024-03-27

**Authors:** Anthony Gaidici, Youssef Galal, Arjun Vohra, Evan S Lederman, Anup Shah

**Affiliations:** 1 Medicine, University of Arizona College of Medicine-Phoenix, Phoenix, USA; 2 Orthopaedic Surgery, Banner University Medical Center-Phoenix, Phoenix, USA

**Keywords:** rct, rotator cuff tear, ultrasound, rcr, daa, dermal allograft augmentation, rotator cuff repair

## Abstract

Introduction

Arthroscopic advancements have notably improved rotator cuff repair, yet the healing of tendons and retear risks still pose significant challenges, especially with massive and large tears. These larger tears have shown a quadruple increase in retear rate when the tear size grows from 1-3 cm. The prevalent cause of failure, particularly in tears with fatty degeneration, is suture tearing. As such, techniques that enhance tendon healing are pivotal due to their correlation with improved functional outcomes. Dermal allograft augmentation (DAA) is one method that has demonstrated improvements in rotator cuff repair biomechanics, as well as the promotion of vascularization and neotendon formation. This study evaluates the efficacy of DAA in the postoperative healing of large and massive rotator cuff tears, assessed through patient-reported outcomes and functional range of motion.

Methods

This retrospective study reviewed a single surgeon’s database, selecting patients who underwent arthroscopic surgery for full-thickness rotator cuff tears measuring at least 3 cm, or those with maximum stage II fatty infiltration of the supraspinatus or infraspinatus muscle on MRI. We used three validated patient-reported outcome (PRO) measures, American Shoulder and Elbow Surgeon Score (ASES), Single Assessment Numeric Evaluation (SANE), and Simple Shoulder Test (SST), alongside a range of motion metrics, external rotation (ER), and forward flexion (FF). These were recorded pre-operatively and, at minimum, one year post-operatively, with a standard student t-test employed for comparative analysis.

Results

Out of 18 total patients, the average age was 61 years, and 83% were male. The cause of injury was mostly traumatic in nature (n=11), with the remainder being chronic (n=7). All three PROs (n=11) demonstrated a statistically significant increase in score including ASES (p=0.003), SANE (p=0.004), and SST (p=0.039). External rotation also significantly improved pre- to post-operative function (46 vs 58, p=0.049). Three patients (17%) suffered from a retear within three months of their rotator cuff repair. Two patients (11%) required a reverse shoulder arthroplasty within a year.

Conclusion

Our study demonstrated that DAA in rotator cuff repair yielded notable functional and clinical advancements, with a moderately low retear rate as confirmed by ultrasound. These outcomes suggest that DAA is a promising intervention for large and historically difficult rotator cuff tears, offering significant implications for future treatment protocols.

## Introduction

Every year in the United States, approximately 98 per 100,000 people will undergo rotator cuff repair, making rotator cuff tears among the most prevalent causes of shoulder pain and disability that are treated by orthopedic surgeons [1]. Rotator cuff injury is the most common tendon injury seen in adults and can be caused by either acute injury or chronic degeneration [2]. Statistically, nearly 30% of adults over 60 years old have had a rotator cuff tear, and approximately 62% of adults over 80 years old have had a rotator cuff tear [3]. Despite the advancement in arthroscopic techniques, adequate tendon healing and the risk of retears remain a significant complication in the efficacy of rotator cuff repair [3]. Results for rotator cuff repair can vary significantly, especially when assessed through imaging. A systematic review published in 2015 found the mean rotator cuff retear rate to be nearly 27% after 24 months post-surgery [4].

Increasing tear size, increasing retraction, and fatty infiltration are the primary factors associated with decreased rates of tendon healing [5-7]. The retear rate, for example, increases four-fold when the initial tear size increases from one to three cm [7]. For this reason, large and massive rotator cuff tears pose a unique challenge to orthopedic surgeons. It is well-established that the most common mode of failure in large and massive rotator cuff tears, especially those with fatty degeneration infiltration, is tendon tearing through the suture [8].

In our study, massive rotator cuff tear refers to a tear involving two or more rotator cuff tendons with a tear size equal to or greater than 3 cm in both the anteroposterior and mediolateral directions, with retraction of the tendons to the glenoid rim in either the coronal or axial plane. Increased retear rates in large and massive rotator cuff tears, as well as the known association between tendon healing and functional outcome, have contributed to the need to search for techniques that can improve tendon healing in these patients [9-12]. Dermal allograft augmentation (DAA) has been shown to improve the biomechanics of rotator cuff repair, and case reports have described the capability for vascularization and neotendon formation [13,14]. One study of 42 patients suggested that DAA can improve healing [12]. However, repairs were limited to single-row constructs, which may have lower healing than double-row constructs.

The primary outcome of this study is to evaluate the postoperative healing of large and massive rotator cuff tears with dermal allograft augmentation. To achieve this, a standardized patient-reported outcome (PRO) scoring system, functional outcomes through a range of motion, and imaging by ultrasound were utilized.

## Materials and methods

**Figure 1:**
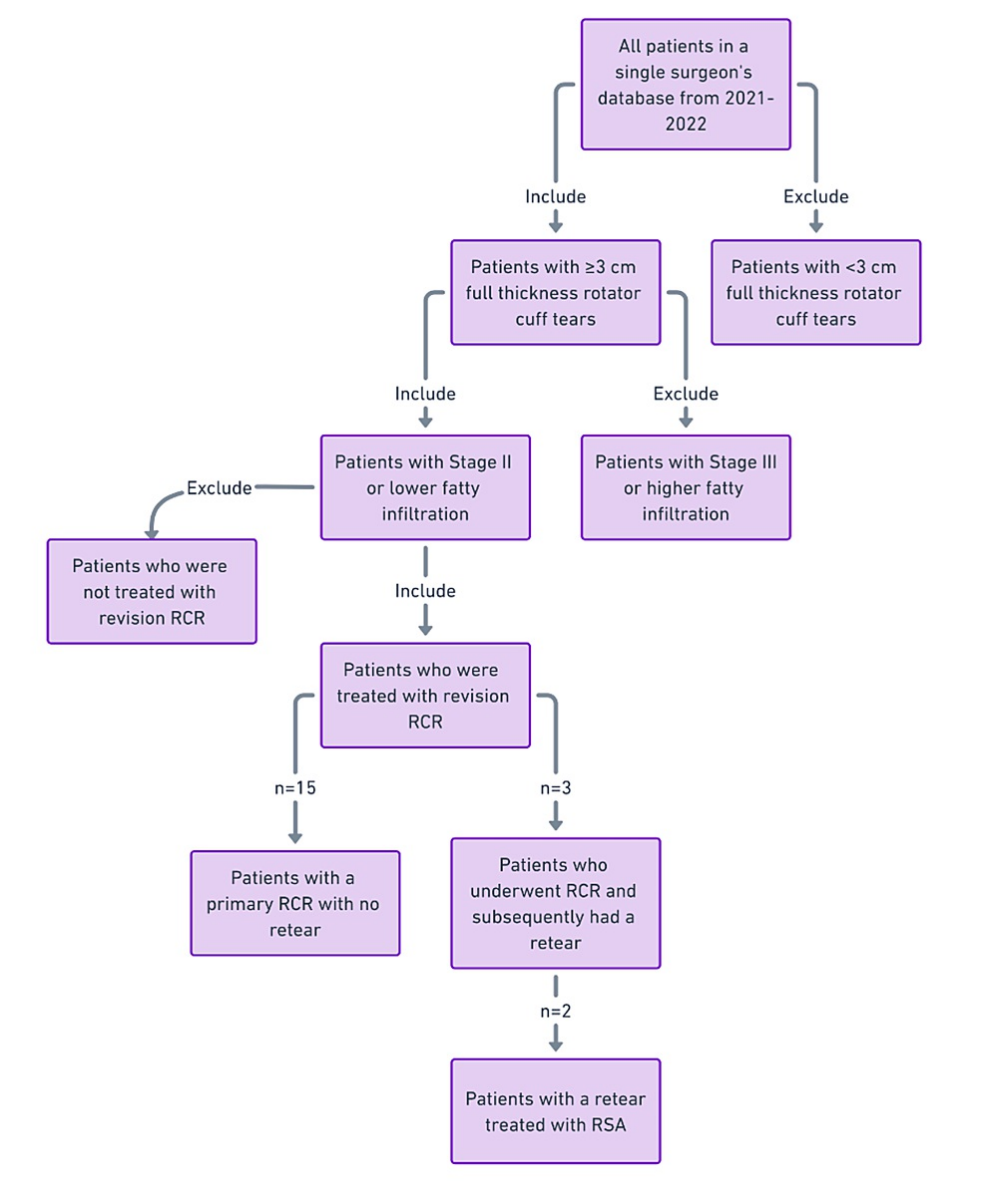
Flowchart depicting the patient selection process

A retrospective review of a single-surgeon database was performed. Patients who received arthroscopic surgery with allograft augmentation using the CuffMend^TM^ system by Arthrex, FL, USA, from 2021-2023 included the following criteria: full-thickness rotator cuff tear with a tear size equal to or greater than 3 cm in either the anterior-posterior or the medial-lateral dimension, stage II fatty infiltration or lower of the supraspinatus/infraspinatus muscle as determined by Goutallier classification based on MRI, and revision rotator cuff repair. A total of 18 patients were included in the case series, which included both primary and revision cases (Figure 1). Three validated patient-reported outcomes scores were utilized as a surrogate for clinical outcomes including: American Shoulder and Elbow Surgeon Score (ASES), Single Assessment Numeric Evaluation Score (SANE), and Simple Shoulder Test (SST). Two range of motion (ROM) values were collected for each patient including external rotation (ER) and forward flexion (FF) in the neutral position. Both ROM and PRO values were collected for each patient pre-operatively and at a minimum of one year follow-up period post-operatively.

**Figure 2:**
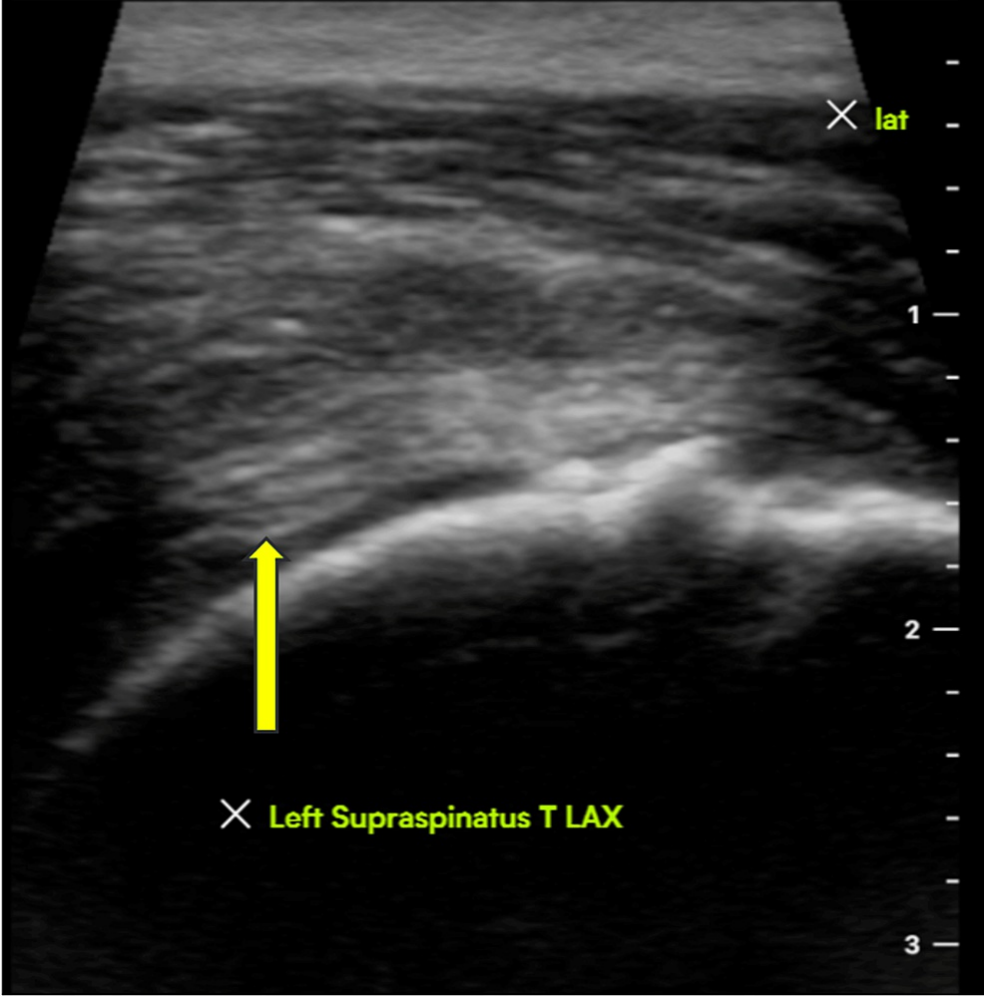
Ultrasound imaging three-month after the procedure (image 1) Ultrasound imaging demonstrating an intact left supraspinatus tendon three months after rotator cuff repair with augmentation. LAX = long axis view.

**Figure 3:**
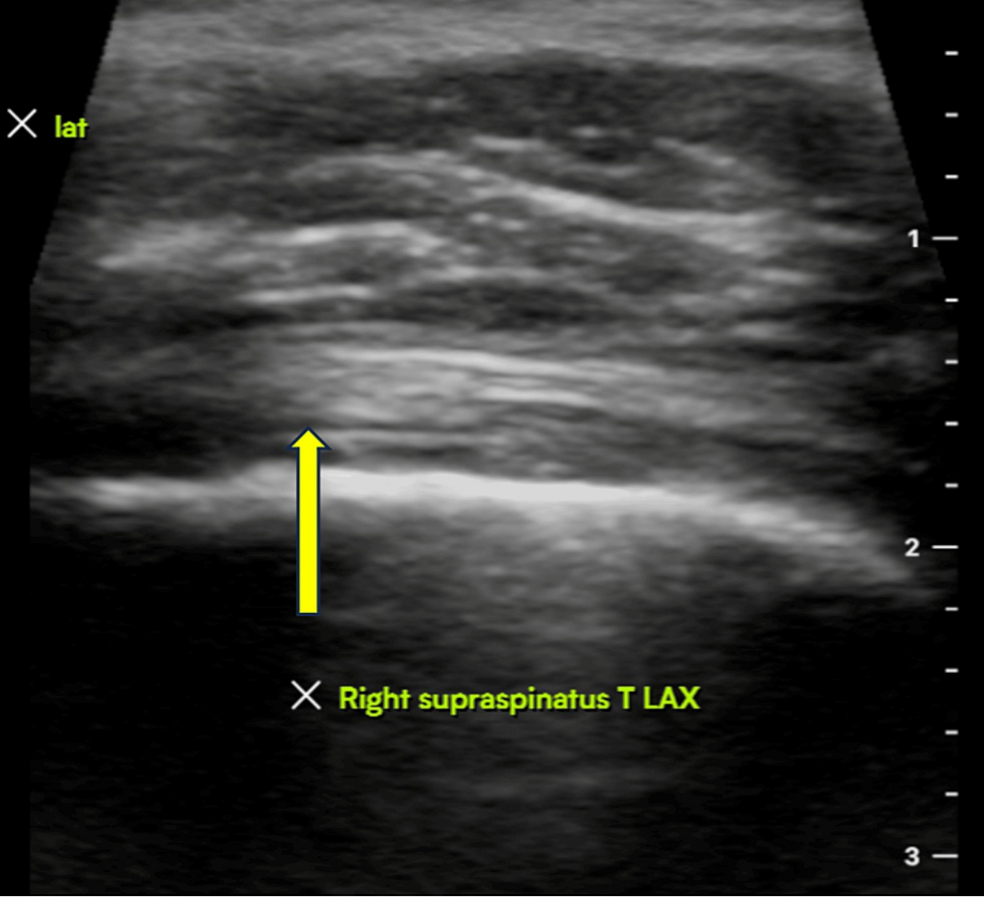
Ultrasound imaging three months after the procedure (image 2) Ultrasound imaging demonstrating an intact right supraspinatus tendon three months after rotator cuff repair with augmentation.

The pre-operative and post-operative scores for functional and clinical outcomes were compared using a standard student t-test. All patients had a postoperative visit within two weeks and were instructed to remain in a sling for four more weeks. Physical therapy was started after four weeks with gentle escalating ROM exercises. The postoperative protocol included six total weeks in a sling, starting passive ROM (PROM) at week 4, starting active ROM (AROM) at week 6, and once 80% of motion obtained compared to contralateral shoulder, strengthening training was commenced, but not sooner than three months. Ultrasounds were obtained within 12 months for evaluation of rotator cuff integrity in asymptomatic and symptomatic patients for comparison purposes (Figures 2, 3).

## Results

The average patient age was 61 years, with 83% male and 17% female patients. The average length of follow-up was 14 months, with a range of three to 21 months, with six patients not following up at or after one year post-operation. The cause of injury was mostly traumatic in nature (n=11), with the remainder being chronic in cause (n=7). This data is summarized in Table 1.

**Table 1:** Patient demographics

Patient Demographics	
Male	15
Female	3
Mean Age	61.1
Mean BMI	29.8
Right Laterality	11
Left Laterality	7
Traumatic Injury	11
Chronic Injury	7

Postoperatively, there was a statistically significant improvement in all three PRO scores in comparison to the preoperative including ASES (p=0.003, 95% CI of 21.2 to 58.2), SANE (p=0.004, 95% CI of 21.4 to 48.0), and SST (p=0.039, 95% CI of 1.7 to 6.3). External rotation demonstrated a statistically significant difference from pre-operative levels to post-operative function (46 vs. 58, p=0.049, 95% CI of 2.35 to 27.65). Improvements in forward flexion postoperatively were not significant statistically (Table 2).

**Table 2:** Functional and clinical outcomes assessed for patients at a minimum follow-up of one year postoperatively ASES = American Shoulder and Elbow Surgeon (score), SANE = Single Assessment Numeric Evaluation (score), SST = Simple Shoulder Test (score), ROM = range of motion, FF = forward flexion (degrees), ER = external rotation (degrees).

	Pre-Operation (Standard Deviation)	Post-Operation (Standard Deviation)	Percent Difference	p value
ASES	44.1 (24.22)	87.6 (9.58)	98.5%	0.003
SANE	41.7 (20.38)	81.8 (14.3)	96.3%	0.004
SST	5.3 (2.79)	10.3 (1.72)	95.3%	0.039
Active ROM FF	119.7 (53.14)	151.1 (6.9)	26.2%	0.057
Active ROM ER	45.8 (17.52)	57.8 (9.72)	26.1%	0.049

Through ultrasound analysis, three of the 18 patients (17%) included in the case series suffered from a retear, which was subsequently associated with pain for all three patients. All three retears occurred within the first three months post-operatively. The first patient endured a recurrent moderately-sized full-thickness tear of the supraspinatus tendon. However, this patient had previously had multiple rotator cuff repair attempts on the same shoulder with retears occurring every time. The second patient had a complete recurrent tear involving both the infraspinatus and supraspinatus. The final patient endured a recurrent rotator cuff tear as an acute injury suffered during weightlifting. All three patients were managed conservatively, which consisted of treatment with physical therapy, pain medications, as well as rest. Of the patients with no retears, two proceeded with a reverse shoulder arthroplasty within one year of their rotator cuff repair arthroscopy due to recurrent pain.

## Discussion

The results of our study demonstrate that patients receiving rotator cuff augmentation with DAA led to improvements in functional and clinical outcomes as well as a relatively low retear rate as diagnosed by ultrasound. Out of the 18 patients, three had recurrent rotator cuff tears, and two needed a reverse shoulder arthroplasty within a year. This finding is in line with the retear rate found at a two-year follow-up in McElvany et al.’s systemic review on rotator cuff tears [4]. Traditionally, success rates in large and massive rotator cuff tear repairs have been lower than in smaller tear repairs, with high failure rates [15]. The results of this study demonstrate a higher success rate in arthroscopic repair of large to massive rotator cuff tears than the 35-94% that has traditionally been cited in the literature [15]. This suggests that the use of dermal allograft augmentation for these large rotator cuff tears that have been historically hard to manage may hold significant promise in the future.

The utilization of biologics that can improve healing and thickness at the repair site has been studied. There is currently a wide variety of extracellular matrix and polymer patches being studied including allograft rotator cuff, human cadaveric skin, pig and bovine skin, equine pericardium, and porcine intestinal submucosa [16]. Smith et al. conducted a large systemic review following 598 patients with rotator cuff tears treated with allograft or autograft, finding a significant improvement in ASES scores, forward flexion, and external rotation, suggesting a favorable use of dermal allograft as a means of treatment of rotator cuff tear [17]. Another systemic review by Makovicka et al. found similar results [18]. Currently, there is significant literature that supports the use of DAA for rotator cuff repair in general, which includes but is not limited to only the CuffMend system. For instance, Cai et al. conducted a large randomized controlled trial using DAA in 112 patients undergoing RCR and found significantly lower retear rates and higher shoulder scores compared to patients who went only traditional arthroscopic suture bridge repair [19]. A meta-analysis and systemic review of 36 articles similarly found that DAA for RCR was associated with significantly lower retear rates and higher ASES scores than RCR alone [20]. A few older and smaller prospective studies confirm this same trend [12,21-23]. One newer study specifically focused on the CuffMend system by Arthrex, and although the authors noted the system’s higher technical demand, they list the lack of need for additional equipment, the cost reduction compared to similar techniques, the increased biomechanical resistance, and the easy visualization of the correct initial repair of the footprint-tendon interface as its considerable advantages [24]. In our study, we utilized the CuffMend system in the treatment of our patients and were satisfied with the similar advantages it offered us.

Initially, the analysis of rotator cuff repair success was determined primarily through the analysis of functional and clinical outcomes. Recently, postoperative imaging, such as magnetic resonance imaging and ultrasound have become more prevalent to evaluate tendon healing. Badman et al. conducted a small study following 10 patients treated with DAA for rotator cuff tears and found a significant increase in ASES score from 43 to 87 (p=0.005), with a 70% graft failure rate after one year [25]. Another study conducted by Hirahara et al. following 18 patients treated with DAA for rotator cuff tears found an ASES scores improvement from 49.3 to 85.1 (p<0.001), and promisingly found significant allograft thickening and vascularization within one year, with no evidence of reabsorption in the body [26]. An additional small study by Hall et al. found a similar 20-point improvement in ASES scores at two-year follow-up following the use of DAA in RCT repair, with all patients having an intact repair as assessed by ultrasound at two-year follow-up [27]. In our study, ultrasound imaging was utilized as a method for evaluating the healing of the rotator cuff repair in patients, as well as assessing for retears in patients who remained symptomatic. Ultrasound imaging modalities present a unique opportunity to assess rotator cuff integrity post-repair while being quicker and much cheaper than the more widely used MRI imaging technique.

This study has multiple limitations. Firstly, as in all case series, this study is limited by not having a control group that was not treated with rotator cuff augmentation techniques. This makes it difficult to fully assess whether or not the quality and stability of the repair were contributed to by the dermal allograft patch. In addition, the postoperative course for the patients was not fully standardized. Future studies should focus on a prospective approach that directly compares patients with massive and large rotator cuff tears treated with and without dermal allograft augmentation in order to assess the effectiveness of the augmentation in the repair. In addition, in this future study, all patients should receive routine ultrasound imaging at their follow-up visits to ensure all tears are reported.

## Conclusions

DAA is a relatively new surgical technique used for treating large rotator cuff tears. This case series followed patients who had rotator cuff tears treated with DAA and found significant improvements in functional and clinical scores such as ASES, SANE, SST, and external rotation at one-year follow-up. There was a moderately low retear rate in this group. These outcomes reinforce recent findings of DAA as a promising intervention for large and historically difficult rotator cuff tears, offering significant implications for future treatment protocols.

## References

[1] Zhang AL, Montgomery SR, Ngo SS, Hame SL, Wang JC, Gamradt SC (2013). Analysis of rotator cuff repair trends in a large private insurance population. Arthroscopy.

[2] Dang A, Davies M (2018). Rotator cuff disease: treatment options and considerations. Sports Med Arthrosc Rev.

[3] Teunis T, Lubberts B, Reilly BT, Ring D (2014). A systematic review and pooled analysis of the prevalence of rotator cuff disease with increasing age. J Shoulder Elbow Surg.

[4] McElvany MD, McGoldrick E, Gee AO, Neradilek MB, Matsen FA 3rd (2015). Rotator cuff repair: published evidence on factors associated with repair integrity and clinical outcome. Am J Sports Med.

[5] Azar M, Van der Meijden O, Pireau N, Chelli M, Gonzalez JF, Boileau P (2022). Arthroscopic revision cuff repair: do tendons have a second chance to heal?. J Shoulder Elbow Surg.

[6] Kwon J, Kim SH, Lee YH, Kim TI, Oh JH (2019). The rotator cuff healing index: a new scoring system to predict rotator cuff healing after surgical repair. Am J Sports Med.

[7] Duong JK, Lam PH, Murrell GA (2021). Anteroposterior tear size, age, hospital, and case number are important predictors of repair integrity: an analysis of 1962 consecutive arthroscopic single-row rotator cuff repairs. J Shoulder Elbow Surg.

[8] Pashuck TD, Hirahara AM, Cook JL, Cook CR, Andersen WJ, Smith MJ (2021). Superior capsular reconstruction using dermal allograft is a safe and effective treatment for massive irreparable rotator cuff tears: 2-year clinical outcomes. Arthroscopy.

[9] Jackson GR, Bedi A, Denard PJ (2022). Graft augmentation of repairable rotator cuff tears: an algorithmic approach based on healing rates. Arthroscopy.

[10] Omae H, Steinmann SP, Zhao C, Zobitz ME, Wongtriratanachai P, Sperling JW, An KN (2012). Biomechanical effect of rotator cuff augmentation with an acellular dermal matrix graft: a cadaver study. Clin Biomech (Bristol, Avon).

[11] Snyder SJ, Arnoczky SP, Bond JL, Dopirak R (2009). Histologic evaluation of a biopsy specimen obtained 3 months after rotator cuff augmentation with GraftJacket Matrix. Arthroscopy.

[12] Barber FA, Burns JP, Deutsch A, Labbé MR, Litchfield RB (2012). A prospective, randomized evaluation of acellular human dermal matrix augmentation for arthroscopic rotator cuff repair. Arthroscopy.

[13] Cummins CA, Murrell GA (2003). Mode of failure for rotator cuff repair with suture anchors identified at revision surgery. J Shoulder Elbow Surg.

[14] Haque A, Pal Singh H (2018). Does structural integrity following rotator cuff repair affect functional outcomes and pain scores? A meta-analysis. Shoulder Elbow.

[15] Chung SW, Kim JY, Kim MH, Kim SH, Oh JH (2013). Arthroscopic repair of massive rotator cuff tears: outcome and analysis of factors associated with healing failure or poor postoperative function. Am J Sports Med.

[16] Ricchetti ET, Aurora A, Iannotti JP, Derwin KA (2012). Scaffold devices for rotator cuff repair. J Shoulder Elbow Surg.

[17] Smith TJ, Gowd AK, Kunkel J (2021). Clinical outcomes of superior capsular reconstruction for massive, irreparable rotator cuff tears: a systematic review comparing acellular dermal allograft and autograft fascia lata. Arthrosc Sports Med Rehabil.

[18] Makovicka JL, Chung AS, Patel KA, Deckey DG, Hassebrock JD, Tokish JM (2020). Superior capsule reconstruction for irreparable rotator cuff tears: a systematic review of biomechanical and clinical outcomes by graft type. J Shoulder Elbow Surg.

[19] Cai YZ, Zhang C, Jin RL, Shen T, Gu PC, Lin XJ, Chen JD (2018). Arthroscopic rotator cuff repair with graft augmentation of 3-dimensional biological collagen for moderate to large tears: a randomized controlled study. Am J Sports Med.

[20] Bailey JR, Kim C, Alentorn-Geli E (2019). Rotator cuff matrix augmentation and interposition: a systematic review and meta-analysis. Am J Sports Med.

[21] Kim KT, Kim GH, Cha DH, Lee JH, Lee YB (2023). A comparison of clinical outcomes in rotator cuff re-tear patients who had either an arthroscopic primary repair or arthroscopic patch augmentation for large-to-massive rotator cuff tears. Diagnostics (Basel).

[22] Neri BR, Chan KW, Kwon YW (2009). Management of massive and irreparable rotator cuff tears. J Shoulder Elbow Surg.

[23] Gilot GJ, Alvarez-Pinzon AM, Barcksdale L, Westerdahl D, Krill M, Peck E (2015). Outcome of large to massive rotator cuff tears repaired with and without extracellular matrix augmentation: a prospective comparative study. Arthroscopy.

[24] Tauro TM, Wagner KR, DeFroda SF, Muth S, Bodendorfer BM, Verma NN, Cole BJ (2022). Technical note: arthroscopic rotator cuff repair with patch augmentation with acellular dermal allograft. Arthrosc Tech.

[25] Badman BL, Baessler AM, Moor M (2020). Short-term clinical outcomes and comparison of ultrasound versus magnetic resonance imaging of superior capsular reconstruction. Arthrosc Sports Med Rehabil.

[26] Hirahara AM, Andersen WJ, Panero AJ (2019). Ultrasound assessment of the superior capsular reconstruction with dermal allograft: an evaluation of graft thickness and vascularity. Arthroscopy.

[27] Hall T, Danielson K, Brandenburg S, Matelic T (2020). A case series of recurrent myotendinous rotator cuff tears repaired and augmented with dermal allograft: clinical outcomes at two years. J Shoulder Elbow Surg.

